# An Entropy-Based Scale-Free L-Statistic for Exponentiality Testing in Lifetime Data with DMRL Aging

**DOI:** 10.3390/e28070755

**Published:** 2026-07-01

**Authors:** Anfal A. Alqefari

**Affiliations:** Department of Statistics and Operations Research, College of Science, Qassim University, P.O. Box 6644, Buraydah 51482, Saudi Arabia; aa.alqefari@qu.edu.sa

**Keywords:** cumulative residual Tsallis entropy, DMRL aging, exponentiality testing, lifetime data analysis, reliability statistics, scale-free L-statistic, 62G10, 62N05, 62E20, 94A17

## Abstract

This paper introduces an entropy-based scale-free L-statistic for exponentiality testing in lifetime data under decreasing mean residual life (DMRL) aging. The proposed method is derived from a mean-residual-life characterization of the DMRL class through a cumulative residual entropy-type departure functional that quantifies deviations from the constant mean residual life property of the exponential distribution. An L-functional representation of this departure measure is established, leading to an order-statistic-based nonparametric test statistic. To remove the effect of the unknown exponential scale parameter, the statistic is normalized by the sample mean, yielding a scale-invariant testing procedure under the null hypothesis. The exact finite-sample null distribution is obtained using normalized spacings, and asymptotic normality is established through standard L-statistic theory. Monte Carlo simulations under linear failure rate, gamma, and Weibull DMRL alternatives show that the proposed test maintains the nominal significance level and provides competitive power relative to existing DMRL procedures. Real-data applications further illustrate the usefulness of the method as an entropy-based statistical tool for detecting DMRL aging patterns in reliability and lifetime-data analysis.

## 1. Introduction

Entropy-based methods play an important role in modern statistics and data science by providing quantitative tools for measuring uncertainty, information content, dispersion, and departures from reference models. In statistical inference, such measures are particularly useful when the objective is not only to fit a parametric model, but also to assess whether observed data exhibit structural features that are inconsistent with a baseline distribution. For non-negative lifetime data, cumulative residual entropy and its generalized forms are especially relevant because they measure uncertainty directly through the survival function. This survival-based information perspective is natural for exponentiality testing, where the exponential distribution serves as the canonical no-aging model and departures from it may reveal meaningful changes in residual lifetime behavior.

In statistical inference and reliability engineering, analyzing non-negative lifetime data is a fundamental task with direct implications for survival analysis, maintenance planning, and risk assessment. A central inferential question is how the age of a surviving unit affects its remaining lifetime. Among standard reliability measures, the mean residual life (MRL) function offers a particularly interpretable description of aging. Unlike the hazard rate, which measures instantaneous failure risk, the MRL function quantifies the expected additional lifetime conditional on survival to a given age, providing a direct summary of anticipated future performance. Let X be a non-negative lifetime random variable with absolutely continuous distribution function F, density f, and survival function $\bar{F}\left(x\right)=1-F(x),x\geq 0.$ For a unit that has survived up to time t≥0, the residual lifetime is defined as $X_{t}=[X-t|X>t]$. The corresponding MRL function is

$$m\left(t\right)=\frac{\int_{t}^{\infty }\bar{F}\left(x\right)\;dx}{\bar{F}\left(t\right)},\;t>0,$$

whenever $\bar{F}\left(t\right)>0$. This function has been extensively used in reliability, survival analysis, actuarial science, biometry, and related areas because it gives a direct measure of the expected remaining operational lifetime; see, for example, Lai and Xie [1]. In this framework, the exponential distribution serves as the canonical baseline model for “no aging,” since its MRL function is constant. Thus, exponentiality corresponds to the absence of aging in the mean-residual-life sense, making it the standard null hypothesis for testing structural departures in lifetime data.

### 1.1. DMRL Aging and Entropy-Based Departure Measures

The monotonicity of $m\left(t\right)$ defines important aging classes. A lifetime distribution is said to have decreasing mean residual life (DMRL) if $m\left(t\right)$ is non-increasing in t, and increasing mean residual life (IMRL) if $m\left(t\right)$ is non-decreasing. The exponential distribution is the natural boundary model in this framework, since its MRL function is constant. Thus, exponentiality corresponds to the absence of aging in the mean-residual-life sense, whereas departure toward the DMRL class indicates that the expected remaining lifetime decreases with age. This behavior is commonly associated with deterioration, wear, fatigue, and cumulative damage. Testing exponentiality against DMRL alternatives is therefore a fundamental problem in reliability and lifetime-data analysis. The exponential model is attractive as a baseline because of its memoryless property and mathematical simplicity. However, when DMRL aging is present, the exponential assumption may mask important deterioration effects and lead to misleading reliability assessments or suboptimal maintenance decisions. The testing problem considered in this paper is

**H_0_.** 
$|\bar{F}\left(x\right)=e^{-\lambda x},\;x>0,|\;\lambda >0$ *.*

**H_1_.** 
∣F∣ belongs to the DMRL class and is not exponential *.*

The exponential distribution is adopted under H_0_ because it is the unique continuous lifetime model with a constant mean residual life and therefore represents the canonical no-aging boundary between decreasing and increasing mean-residual-life behavior. A Weibull distribution is not used as the null model because, except at shape parameter θ = 1, it already exhibits aging and is consequently more appropriate as an alternative. The linear failure rate (LFR), gamma, and Weibull families, each containing the exponential model at a boundary parameter value and exhibiting DMRL behavior over the parameter ranges considered, are therefore used as representative alternatives in the simulation study (Section 4.1).

Throughout the paper, powers of the survival function are interpreted pointwise; in particular, ${\bar{F}}^{\beta }\left(t\right)$ means ${\left[\bar{F}\left(t\right)\right]}^{\beta }$.

### 1.2. Literature Review and the Research Gap

Several nonparametric tests have been developed for testing exponentiality against DMRL and closely related aging alternatives. The test of Hollander and Proschan [2] initiated a broad class of procedures based on aging inequalities. Further developments used the Total Time on Test transform and related characterizations, including the contributions of Abouammoh and Khalique [3], Klefsjö [4], and Bergman and Klefsjö [5]. Other approaches focused more directly on the MRL function and its monotonicity; for instance, Aly [6] proposed tests for MRL monotonicity, Ahmad [7] introduced a procedure with improved performance, and Lim and Park [8,9] provided further developments. More recent contributions have employed quantile-based, dispersion-based, and moment-inequality arguments, including the works of Fernandez-Ponce et al. [10], Belzunce et al. [11,12], Abu-Youssef [13], Ahmad and Mugdadi [14], Li et al. [15], Lorenzo et al. [16], Anis [17], and Zardasht [18]. Building on these foundations, recent studies have continued to enrich the literature on lifetime data analysis and exponentiality testing through new entropy-based measures, nonparametric procedures, and L-statistic approaches (e.g., Bhattacharyya et al. [19], Mohamed et al. [20], Ebner [21], and Sepehrifar [22]). Despite this substantial literature, relatively few tests combine aging interpretation, entropy-based departure measurement, scale invariance, and tractable distribution theory within a single framework. Existing procedures are commonly built on aging inequalities, total-time-on-test characterizations, residual-life dispersion measures, or moment-type contrasts. Although these approaches have proved useful, they do not directly express departure from exponentiality through an entropy-based functional that is linked to a DMRL characterization and admits an L-functional representation. Recent structural-reliability research has also addressed multi-state systems with dependent components and imprecise parameters [23], illustrating the broader movement toward reliability models that accommodate complex dependence and uncertainty.

To make the methodological positioning transparent, Table 1 compares the proposed procedure with representative families of existing tests and summarizes the specific combination of features contributed by the present study.

### 1.3. Motivation, Novelty, and Paper Organization

Specifically, the area lacking in the current literature is a unified framework that simultaneously combines an aging interpretation, an entropy-based departure measure, scale invariance, and tractable distribution theory. To address this specific gap, this paper develops a unified framework by proposing an entropy-based scale-free L-statistic for testing exponentiality against DMRL aging. The novelty of this paper lies in deriving a scale-free L-statistic from a cumulative residual Tsallis entropy-type functional, which uniquely connects the aging interpretation of the alternative with a computable order-statistic-based statistic, while providing access to exact finite-sample and asymptotic null distribution theory. The construction is derived from a mean-residual-life characterization of the DMRL class and is based on a cumulative residual entropy-type functional that measures deviations from the constant mean residual life property of the exponential distribution. The functional is shown to have an L-functional representation, leading to a nonparametric statistic expressed in terms of order statistics. Normalization by the sample mean removes dependence on the unknown scale parameter and yields a test invariant under positive scale transformations.

The remainder of this paper is organized as follows. Section 2 develops the entropy-based departure functional, its L-functional representation, and the corresponding scale-free statistic. Section 3 establishes the exact finite-sample null distribution and the asymptotic distributional theory. Section 4 reports the Monte Carlo study and compares the proposed procedure with existing DMRL tests under linear failure rate, gamma, and Weibull alternatives. Section 5 presents real-data applications. Section 6 concludes with a summary of the main findings and directions for future research.

For simplicity, throughout the remainder of this paper, we write $g^{a}(x)$ instead of ${\left[g\left(x\right)\right]}^{a}$. Unless stated otherwise, it is assumed that all expectations and integrals appearing in the paper exist and are finite.

For the convenience of the reader, the principal mathematical symbols and scientific abbreviations used throughout the paper are summarized in Table 2.

## 2. A Scale-Free Entropy-Based L-Statistic

The section focuses on constructing the entropy-based departure functional, deriving its L-functional representation, and obtaining the corresponding scale-free sample statistic. Let

$$E=\{F_{\lambda }:F_{\lambda }\left(x\right)=1-e^{-\lambda x},|x\geq 0,|\lambda >0\}$$

be the class of exponential distributions. The testing problem considered in this paper is

$$H_{0}:F\in E\;\mathrm{versus}\;H_{1}:F\in \mathrm{DMRL},|F\notin E.$$

Let $X_{1},\;X_{2},\ldots ,\;X_{n}$ be an independent random sample drawn from the absolutely continuous distribution F. The aim is to construct a statistic that is zero under exponentiality and tends to take positive values when F belongs to the DMRL class but is not exponential.

### 2.1. Entropy-Based Departure Functional for DMRL Aging

To connect the proposed statistic with a cumulative residual entropy-type quantity, consider the proportional hazards transform generated by the baseline survival function $|\bar{F}$. For β > 0, let $X_{\beta }$ be a random variable with survival and density functions ${\bar{F}}_{\beta }\left(t\right)={\bar{F}}^{\beta }\left(t\right),$ and $f_{\beta }\left(t\right)=\beta f\left(t\right){\bar{F}}^{\beta -1}\left(t\right),t\geq 0,$ respectively. The weighted mean of the MRL function with respect to this transformed distribution is

$$E\left[m\left(X_{\beta }\right)\right]=\beta \int_{0}^{\infty }m\left(t\right)f\left(t\right){\bar{F}}^{\beta -1}\left(t\right)\;dt.$$

Using the representation of m(t) given in Section 1 and applying Fubini’s theorem, for β>0 and β≠1 we obtain

(1) $$\begin{array}{c} E\left[m\left(X_{\beta }\right)\right]=\beta \int_{0}^{\infty }f\left(t\right){\bar{F}}^{\beta -2}\left(t\right)\left(\int_{t}^{\infty }\bar{F}\left(x\right)\;dx\right)dt.\\ E\left[m\left(X_{\beta }\right)\right]=\beta \int_{0}^{\infty }\bar{F}\left(x\right)\left(\int_{0}^{x}f\left(t\right){\bar{F}}^{\beta -2}\left(t\right)\;dt\right)dx.\\ E\left[m\left(X_{\beta }\right)\right]=\frac{\beta }{\beta -1}\left[\int_{0}^{\infty }\bar{F}\left(x\right)\;dx-\int_{0}^{\infty }{\bar{F}}^{\beta }\left(x\right)\;dx\right]. \end{array}$$

The interchange of the order of integration in Equation (1) is justified by Tonelli’s theorem for the non-negative integrand, together with the finiteness assumptions required for the resulting quantities. In particular, when β > 1, the finite-mean condition μ < ∞ is sufficient because $\bar{F}$(x)^β ≤ $\bar{F}$(x). For 0 < β < 1, we additionally assume ∫_0_∞ $\bar{F}$(x)^β dx < ∞. These conditions ensure that the cumulative residual Tsallis entropy and the departure functional are finite.

$$\int_{0}^{x}f\left(t\right){\bar{F}}^{\beta -2}\left(t\right)\;dt=\frac{1-{\bar{F}}^{\beta -1}\left(x\right)}{\beta -1},\;\;x>0,$$

with the limiting case β = 1 giving $-\log\bar{F}(x).$ The limiting case β=1 corresponds to the usual cumulative residual entropy form. For β>0 and β≠1, the cumulative residual Tsallis entropy (CRTE) of order β is defined by

$$S_{\beta }\left(X\right)=\frac{1}{\beta -1}\left[\int_{0}^{\infty }\bar{F}\left(x\right)\;dx-\int_{0}^{\infty }{\bar{F}}^{\beta }\left(x\right)\;dx\right].$$

Consequently, Equation (1) can be written compactly as $E\left[m\left(X_{\beta }\right)\right]=\beta S_{\beta }\left(X\right)$. This quantity was introduced by Rajesh and Sunoj [24] by replacing the density in the classical Tsallis-Havrda-Charvat entropy with the survival function. The underlying generalized entropy of order β goes back to Havrda and Charvat [25] and was later popularized in statistical physics by Tsallis [26].

If X is DMRL, then m(t) ≤ m(0) = μ for all t≥0, which yields

$$E\left[m\left(X_{\beta }\right)\right]\leq \int_{0}^{\infty }\bar{F}\left(x\right)\;dx,$$

for β>0 and β≠1. This observation, together with Equation (1), motivates the following departure functional:

(2) $$\begin{array}{r} T_{\beta }\left(F\right)=\frac{\beta }{\beta -1}\int_{0}^{\infty }{\bar{F}}^{\beta }\left(x\right)\;dx-\frac{1}{\beta -1}\int_{0}^{\infty }\bar{F}\left(x\right)\;dx,\;\beta >0,|\beta \neq 1. \end{array}$$

Under $H_{0}$, $T_{\beta }\left(F\right)=0$, whereas under strictly DMRL alternatives the population departure functional is positive. Thus, $T_{\beta }\left(F\right)$ provides a natural measure of departure from exponentiality in the direction of DMRL aging.

**Example** **1.** *Let* X *follow a Weibull distribution with survival function* $\bar{F}\left(x\right)=e^{-x^{\theta }},x>0,|\theta >0.$ *Then*

$$\int_{0}^{\infty }{\bar{F}}^{\beta }\left(x\right)\;dx=\int_{0}^{\infty }e^{-\beta x^{\theta }}\;dx=\beta^{-1/\theta }\Gamma \left(1+\frac{1}{\theta }\right).$$

*Substituting this expression into Equation (2) gives*

$$T_{\beta }\left(F\right)=\Gamma \left(1+\frac{1}{\theta }\right)\frac{\beta^{\left(\theta -1\right)/\theta }-1}{\beta -1},\;\;\beta >0,|\beta \neq 1.$$

*When* θ* = 1, the distribution reduces to the exponential model and* $T_{\beta }\left(F\right)=0$*. When* θ>1*, the Weibull distribution is strictly DMRL. In this case, *$p=\left(\theta -1\right)/\theta$ *belongs to (0, 1), and the sign of* $\beta^{p}-1$* is the same as the sign of* β−1 *for every* β>0, β≠1*. Hence the above ratio is positive, confirming that* $T_{\beta }\left(F\right)$ *detects departures from exponentiality toward DMRL aging in this representative family.*

### 2.2. L-Functional Representation of the Departure Measure

The departure functional in Equation (2) admits a useful L-functional representation. By integration by parts,

$$\int_{0}^{\infty }{\bar{F}}^{\beta }\left(x\right)\;dx=\int_{0}^{\infty }x\beta f\left(x\right){\bar{F}}^{\beta -1}\left(x\right)\;dx.$$

Consequently, Equation (2) may be written as

(3) $$T_{\beta }\left(F\right)=\int_{0}^{\infty }xJ_{\beta }\left(F\left(x\right)\right)\;dF\left(x\right),$$

where the score function is

(4) $${\;J}_{\beta }\left(u\right)=\frac{\beta^{2}{\left(1-u\right)}^{\beta -1}-1}{\beta -1},\;0<u<1,|\beta >0,|\beta \neq 1.$$

The factor $\beta^{2}$ appears because the coefficient β in Equation (2) is combined with the derivative of ${\bar{F}}^{\beta }(x)$ under integration by parts. Equivalently, if *Q*(*u*) = *F*^−1^(*u*) denotes the quantile function, then

$$T_{\beta }\left(F\right)=\int_{0}^{1}Q\left(u\right)J_{\beta }\left(u\right)\;du.$$

This representation places the proposed functional within the class of L-functionals and provides a direct route to a sample statistic based on order statistics. The score function in Equation (4) determines how observations from different parts of the distribution contribute to the departure measure.

### 2.3. Sample L-Statistic and Scale-Free Formulation

Let $F_{n}$ be the empirical distribution function and let $X_{\left(1\right)},\;X_{\left(2\right)},\ldots ,\;X_{\left(n\right)}$ denote the ordered sample. Substituting $F_{n}$ for F in Equation (3) gives the empirical L-statistic

$${\hat{T}}_{\beta }=\int_{0}^{\infty }xJ_{\beta }\left(F_{n}\left(x\right)\right)\;dF_{n}\left(x\right)=\frac{1}{n}\sum_{i=1}^{n}J_{\beta }\left(\frac{i}{n}\right)X_{\left(i\right)}.$$

Because $T_{\beta }\left(F\right)$ is not scale invariant, we define the population scale-free functional by

$$T_{\beta }^{⋆}\left(F\right)=\frac{T_{\beta }\left(F\right)}{\mu }.$$

In practice, μ is replaced by the sample mean. This yields the scale-free statistic

(5) $${\hat{T}}_{\beta }^{⋆}=\frac{{\hat{T}}_{\beta }}{\bar{X}}.$$

Equation (5) follows directly from the plug-in principle. Since $T_{\beta }^{⋆}\left(F\right)=T_{\beta }\left(F\right)/\mu$, replacing $T_{\beta }\left(F\right)$ by ${\hat{T}}_{\beta }$ and μ by $\bar{X}$ gives

$${\hat{T}}_{\beta }^{⋆}=\frac{{\hat{T}}_{\beta }}{\bar{X}}=\frac{\sum_{i=1}^{n}J_{\beta }\left(i/n\right)X_{\left(i\right)}}{\sum_{i=1}^{n}X_{i}}.$$

This representation also makes the scale invariance transparent: multiplying every observation by a positive constant multiplies both the numerator and the denominator by the same constant, leaving ${\hat{T}}_{\beta }^{⋆}$ unchanged.

**Remark** **1.** *From a practical reliability perspective, the mathematical formulation of the proposed statistic has a direct physical interpretation. The empirical statistic* ${\hat{T}}_{\beta }$ *is a linear combination of order statistics (sorted failure times). The score function *$J_{\beta }\left(\frac{i}{n}\right)$ *acts as a weighting mechanism that determines how much emphasis the test places on different portions of the lifetime distribution. By adjusting the tuning parameter β, a reliability engineer can control the test’s sensitivity: smaller values of β place relatively more weight on early-to-mid life failures, while larger values of β emphasize the tail of the distribution, making the test more sensitive to severe, late-stage wear-out phenomena. Furthermore, the scale-free statistic* ${\hat{T}}_{\beta }^{⋆}$ *can be practically interpreted as a normalized ‘aging index.’ By dividing the departure measure by the sample mean, the statistic quantifies the severity of the deterioration (DMRL aging) relative to the average lifetime of the component, rendering the test completely independent of the unit of measurement (e.g., hours, cycles, or miles).*

### 2.4. Positivity of the Departure Functional Under DMRL Alternatives

The following result justifies the use of large positive values of the statistic as evidence against exponentiality in favor of DMRL alternatives.

**Theorem** **1.** *Assume that* F *is absolutely continuous, has finite mean μ, and that T_β(F) is finite. If F is strictly DMRL and is not exponential, then*

$$T_{\beta }\left(F\right)>0,\;\beta >0,|\beta \neq 1.$$

A complete proof, including the strict-inequality argument and all required integrability conditions, is provided in Supplementary File.

Consequently, ${\hat{T}}_{\beta }^{⋆}>0$, and the corresponding sample statistic is consistent for a positive population departure under standard regularity conditions.

Under the DMRL alternative, the statistic is expected to take relatively large positive values. Hence, the proposed level-α test rejects H_0_ for large values of the scale-free statistic, namely when

$${\hat{T}}_{\beta }^{⋆}>c_{\alpha },$$

where $c_{\alpha }$ is chosen so that the test has significance level α under the exponential null hypothesis.

## 3. Distributional Theory of the Scale-Free Statistic

This section develops the exact finite-sample null distribution and the large-sample distribution of the scale-free statistic introduced in Section 2. We retain the notation of Section 2 and write $J_{\beta }$ for the score function, ${\hat{T}}_{\beta }$ for the empirical L-statistic, and ${\hat{T}}_{\beta }^{⋆}={\hat{T}}_{\beta }/\bar{X}$ for its scale-free version. Since the statistic is scale invariant, all null distributional results are independent of the unknown exponential rate parameter.

### 3.1. Exact Finite-Sample Null Distribution

Let $X_{\left(1\right)}<X_{\left(2\right)}<\ldots <X_{\left(n\right)}$ denote the order statistics of an independent sample from the exponential distribution with rate λ>0, and set $X_{\left(0\right)}=0$. Define the normalized spacings

$$Y_{j}=\left(n-j+1\right)\{X_{\left(j\right)}-X_{\left(j-1\right)}\},\;\;j=1,\ldots ,n.$$

Under $H_{0}$, the random variables $Y_{1},Y_{2},\ldots ,Y_{n}$ are independent exponential random variables with common rate λ. Moreover, the order statistics admit the representation

(6) $$X_{\left(i\right)}=\sum_{j=1}^{i}\frac{Y_{j}}{n-j+1},i=1,\ldots ,n.$$

Using (6) in the definition of the empirical statistic and noting that the sample mean can be written in terms of the normalized spacings, the scale-free statistic can be expressed as a ratio of two linear combinations of independent exponential variables:

$$\begin{array}{c} {\hat{T}}_{\beta }^{⋆}=\frac{\sum_{j=1}^{n}c_{j,n}\left(\beta \right)Y_{j}}{\sum_{j=1}^{n}Y_{j}},\\ c_{j,n}\left(\beta \right)=\frac{1}{n-j+1}\sum_{i=j}^{n}J_{\beta }\left(\frac{i}{n}\right),\;\;j=1,\ldots ,n. \end{array}$$

This representation is useful because the exponential rate λ cancels from the ratio. Hence the null distribution depends only on n, β, and the deterministic coefficients $c_{j,n}\left(\beta \right)$. The normalized-spacing representation is closely related to the exact null-distribution approach used by Langenberg and Srinivasan [27].

The following result provides the exact finite-sample distribution. It is stated for the case of pairwise distinct coefficients; when repeated or nearly repeated coefficients occur, critical values can be obtained by null simulation as described below.

**Theorem** **2.** *Assume that* $H_{0}$ *holds and that the coefficients* $c_{1,n}\left(\beta \right),\ldots ,c_{n,n}\left(\beta \right)$ *are pairwise distinct. Then the exact finite-sample distribution function of the scale-free statistic is given by*

(7) $$P\left\{{\hat{T}}_{\beta }^{⋆}\leq x\right\}=\sum_{j=1}^{n}\frac{max\{x-c_{j,n}\left(\beta \right),0{\}}^{n-1}}{\prod_{k\neq j}\left(c_{k,n}\left(\beta \right)-c_{j,n}\left(\beta \right)\right)}$$

*with support* $\left[{min}_{j}c_{j,n}\left(\beta \right),{max}_{j}c_{j,n}\left(\beta \right)\right]$.

In Equation (7), $\prod_{k\neq j}\{c_{k,n}\left(\beta \right)-c_{j,n}\left(\beta \right)\}$ is an ordinary first-power product. No exponent j is attached to the product term. The normalized-spacing/Dirichlet derivation is given in Supplementary File.

A complete proof, including the transformation to normalized spacings and the treatment of the pairwise-distinct coefficient condition, is provided in Supplementary File.

**Remark** **2.** *Formula (7) provides an exact finite-sample null distribution. However, for larger sample sizes, evaluating the exact formula can become computationally demanding and numerically unstable, particularly when the coefficients* $c_{j,n}\left(\beta \right)$ *are numerically close or repeated. In such cases, and for routine applications with moderate to large n, critical values should be obtained by direct Monte Carlo simulation under the standard exponential distribution. Because the proposed statistic is strictly scale-invariant, simulating from the standard exponential distribution entails no loss of generality. This simulation-based approach is computationally trivial, highly efficient, and avoids the numerical complexities of the exact formula, even for very large sample sizes.*

### 3.2. Asymptotic Normality

We next derive the large-sample distribution of the proposed statistic. Let

$$T_{\beta }\left(F\right)=\int_{0}^{1}Q\left(u\right)J_{\beta }\left(u\right)\;du,$$

where Q(u) = F^−1^(u) is the quantile function. The corresponding empirical functional is an L-statistic. For the parameter range used in the numerical study (β > 1), the score function Jβ is bounded and of bounded variation on (0, 1). We assume that F is continuous, has a finite second moment, and possesses a continuous density f that is strictly positive on the relevant support. We also require finiteness of the double-integral expression defining the asymptotic variance. Under these conditions, the smooth-weight L-statistic asymptotic normality result of Stigler [28] applies.

**Theorem** **3.** *Suppose that F is continuous, E[X^2^] < ∞, f is continuous and strictly positive on the relevant support, Jβ is bounded and of bounded variation on (0, 1), and the asymptotic-variance integral below is finite. Then, as n → ∞,*

$$\sqrt{n}\;\{{\hat{T}}_{\beta }-T_{\beta }\left(F\right)\}\overset{d}{\to }N\{0,\sigma_{\beta }^{2}\left(F\right)\},$$

*where*

$$\sigma_{\beta }^{2}\left(F\right)=\int_{0}^{1}\int_{0}^{1}J_{\beta }\left(u\right)J_{\beta }\left(v\right)\frac{min\left(u,v\right)-uv}{f\left(Q\left(u\right)\right)f\left(Q\left(v\right)\right)}\;du\;dv.$$

Theorem 3 follows directly from the classical asymptotic theory of L-statistics, since the empirical functional is a linear combination of order statistics with deterministic scores generated by $J_{\beta }$; see Stigler [28].

The scale-free statistic involves the additional normalization by the sample mean. The following result is obtained by combining Theorem 3 with the central limit theorem for the sample mean and applying the delta method.

**Theorem** **4.** *Under the conditions of Theorem 3, and assuming* $\mu =E\left(X\right)>0$*,*

$$\sqrt{n}\;\{{\hat{T}}_{\beta }^{⋆}-T_{\beta }^{⋆}\left(F\right)\}\overset{d}{\to }N\{0,\tau_{\beta }^{2}\left(F\right)\},\;\;T_{\beta }^{⋆}\left(F\right)=\frac{T_{\beta }\left(F\right)}{\mu },$$

*where*

(8) $$\tau_{\beta }^{2}\left(F\right)=\frac{\sigma_{\beta }^{2}\left(F\right)}{\mu^{2}}+\frac{T_{\beta }^{2}\left(F\right)}{\mu^{4}}\sigma_{X}^{2}-\frac{2T_{\beta }\left(F\right)}{\mu^{3}}\sigma_{\beta \mu }\left(F\right).$$

*Here,* $\sigma_{X}^{2}=Var\left(X\right)$*, and the asymptotic covariance between the empirical L-statistic and the sample mean is explicitly*

$$\sigma_{\beta \mu }\left(F\right)=\int_{0}^{1}\int_{0}^{1}J_{\beta }\left(u\right)\;\frac{min\left(u,v\right)-uv}{f\{Q\left(u\right)\}f\{Q\left(v\right)\}}\;du\;dv.$$

Under the exponential null hypothesis, $T_{\beta }\left(F\right)=0$, so the terms in Equation (8) involving $T_{\beta }\left(F\right)$ vanish and the variance reduces to the null expression given below

$$\tau_{\beta ,0}^{2}=\frac{\sigma_{\beta ,0}^{2}}{\mu^{2}},$$

where $\sigma_{\beta ,0}^{2}$ is evaluated under the exponential model. Because the statistic is scale free, $\tau_{\beta ,0}^{2}$ does not depend on the unknown exponential rate parameter. Thus, one may set λ=1 when computing asymptotic critical values or carrying out null simulations. For large samples, a level-α test rejects $H_{0}$ in favor of DMRL alternatives for large positive values of the standardized statistic, namely when

$$\frac{\sqrt{n}\;{\hat{T}}_{\beta }^{⋆}}{{\hat{\tau }}_{\beta ,0}}>z_{1-\alpha },$$

where $z_{1-\alpha }$ is the $\left(1-\alpha \right)$-quantile of the standard normal distribution and ${\hat{\tau }}_{\beta ,0}$ is a consistent estimator, or a Monte Carlo approximation, of the null standard deviation. For small and moderate sample sizes, the exact distribution in Theorem 2 or simulated null critical values are preferable.

## 4. Simulation Study

This section evaluates the finite-sample performance of the entropy-based scale-free statistic developed in Section 2 and supported by the distributional results in Section 3. The simulation study has three objectives. First, it examines the sensitivity of the proposed statistic to the tuning parameter β under representative DMRL alternatives. Second, it assesses empirical size under the exponential null model. Third, it compares the proposed test with established nonparametric procedures for testing exponentiality against DMRL aging. All rejection probabilities reported in this section are estimated by Monte Carlo simulation. The nominal significance level is α=0.05. Because the proposed statistic is invariant under positive scale transformations, the scale parameter of each model is fixed without loss of generality. Thus, the empirical behavior of the test is governed by the sample size, the alternative shape parameter, and the tuning parameter β.

### 4.1. Simulation Design and Alternative Models

For each combination of sample size, model, and shape parameter, B = 5000 independent samples were generated. Critical values were obtained under the standard exponential distribution. This entails no loss of generality because the proposed statistic is scale free. The empirical rejection probability was then computed as the proportion of simulated samples for which the observed statistic exceeded the corresponding null critical value. The study considers three lifetime families commonly used in reliability and survival analysis: the linear failure rate (LFR), gamma, and Weibull distributions. These alternatives provide different degrees and patterns of departure from the exponential model within the DMRL class. The LFR model represents a smooth quadratic departure in the cumulative hazard; the gamma and Weibull models provide flexible shape-controlled departures from the exponential boundary. The survival functions used in the simulation are summarized in Table 3.

The parameter values for the alternative distributions were deliberately chosen to represent varying degrees of departure from the exponential boundary within the DMRL class. For the Weibull and Gamma models, the exponential distribution corresponds to a shape parameter of θ=1. By selecting values slightly greater than 1 (e.g., θ∈{1.1, 1.3} for Weibull and θ∈{1.2, 1.6} for Gamma), the simulation evaluates the test’s power against weak DMRL departures. Conversely, larger values (e.g., θ∈{1.5, 1.9} for Weibull and up to 2.6 for Gamma) are utilized to assess performance under strong, severe wear-out departures. A similar graded logic was applied to the LFR model, where θ=0 represents the exponential boundary. This systematic selection ensures a comprehensive evaluation of the test’s sensitivity across a wide spectrum of DMRL aging intensities.

### 4.2. Sensitivity to the Tuning Parameter

The proposed statistic depends on the tuning parameter β, which determines the weighting induced by the entropy-based L-functional. Table 4, Table 5 and Table 6 report empirical powers for β = 1.5, 2.0, …, 5.0 under the LFR, gamma, and Weibull alternatives, respectively. These results assess the stability of the test over a reasonable range of weighting schemes and guide the choice of β in the comparative study. For a fixed β, empirical power generally increases with the sample size and with the distance of the shape parameter from the exponential boundary. No single value of β is uniformly optimal across all alternatives. Smaller values of β tend to perform well under LFR alternatives, whereas larger values may be advantageous for some gamma and Weibull configurations. This behavior is consistent with the role of β in changing the relative weight assigned to different parts of the ordered sample.

For the benchmark comparisons, a single prespecified value β = 2.0 is used for the proposed test under all three alternative families. This family-neutral choice is made before examining the comparative power tables and avoids selecting β separately for LFR, gamma, and Weibull alternatives. The remaining β values in Table 4, Table 5 and Table 6 are retained only as a sensitivity analysis. In applications with no reliable prior information, β = 2.0 is therefore the recommended default, while a sensitivity analysis may be reported as a secondary robustness check.

Based on the simulation findings, the following practical guidelines are suggested:

- For applications where the alternative is expected to be close to exponential (weak DMRL departures), smaller values of β (1.5–2.0) are recommended as they provide more stable power near the null boundary.
- For scenarios where strong DMRL aging is anticipated (e.g., wear-out failures in mechanical systems), moderate to larger values (3.0–4.0) may be preferred.
- For exploratory analysis where the degree of departure is unknown, a sensitivity check over β ∈ {1.5, 2.0, 3.0, 4.0} is advisable. If the test decision is consistent across this range, one can have confidence in the conclusion.
- When prior information about the failure mechanism is available (e.g., from engineering knowledge), this can inform the choice of β by selecting weights that emphasize the relevant parts of the lifetime distribution.
- As a default choice for routine application without prior information, β = 2.0 provides a reasonable compromise across the alternatives studied.

### 4.3. Empirical Size and Benchmark Tests

To place the proposed procedure in context, it is compared with several established DMRL tests from the literature. The benchmark statistics used in the comparison are summarized in Table 7. These include procedures based on aging inequalities, MRL monotonicity, dispersion-type characterizations, and related nonparametric contrasts. The proposed statistic is then evaluated under the exponential null model before its power is compared under the three DMRL alternative families. Table 8 reports empirical Type I error probabilities at the nominal level α=0.05. The values are close to the target level across the considered sample sizes. This indicates that the proposed procedure has adequate size control under the exponential null model, which is an essential requirement before interpreting its power under DMRL alternatives. The small deviations from 0.05 are consistent with Monte Carlo variability.

The Type I error rates in Table 8 are close to the nominal level across the considered sample sizes for the prespecified default β = 2.0. The entries were estimated using B = 5000 independent replications. Since an empirical size is a binomial proportion, its standard error at α = 0.05 is approximately sqrt[0.05(0.95)/5000] = 0.0031, giving an approximate 95% Monte Carlo interval [0.044, 0.056]. The proposed-test rejection rates range from 0.0468 to 0.0530 and therefore lie within this interval, confirming that the small deviations from 0.05 are compatible with Monte Carlo sampling variability.

### 4.4. Power Comparison Under DMRL Alternatives

Table 9, Table 10 and Table 11 present comparative power results under the LFR, gamma, and Weibull alternatives. To ensure a balanced comparison with benchmark procedures, the proposed test uses the same prespecified value β = 2.0 in every table; no alternative-family-specific tuning is used. The results are reported over several sample sizes and shape parameters, covering both near-boundary and pronounced DMRL departures. Empirical power increases with sample size and distance from the exponential boundary. The proposed procedure is not uniformly best, but it performs strongly across the three families while retaining scale invariance, an order-statistic representation, and a direct entropy-based interpretation.

Because no single procedure dominates in every scenario, the comparison should be interpreted in terms of overall stability rather than isolated maxima. Table 12 therefore summarizes the average rank of each procedure across all 63 configurations in Table 9, Table 10 and Table 11, with rank 1 assigned to the largest empirical power in each configuration and average ranks used for ties.

The proposed test attains an overall average rank of 2.37, placing second among the twelve procedures, behind S11 (1.78) and ahead of S2 (2.83). Its family-specific average ranks are 2.43 for LFR, 2.52 for gamma, and 2.14 for Weibull alternatives. These results support the more precise conclusion that the proposed method offers consistently strong, though not uniformly dominant, power under a single family-neutral tuning choice.

### 4.5. Summary of Simulation Findings

The simulation study supports the theoretical development of the entropy-based scale-free L-statistic. The proposed procedure maintains the nominal significance level under the exponential null model. Using the same prespecified value β = 2.0 under every alternative family, it achieves an overall average power rank of 2.37 across 63 configurations, second among the twelve compared procedures. This finding indicates stable and competitive performance rather than universal dominance. The sensitivity tables remain useful for understanding how β redistributes power, but the family-neutral benchmark and the default recommendation β = 2.0 provide a practically implementable protocol when the underlying alternative is unknown.

A limitation worth noting is the test’s performance under very weak departures from exponentiality. As seen in the simulation results for shape parameters close to the exponential boundary (e.g., Weibull θ = 1.1, Gamma shape = 1.2), the empirical power is relatively modest, especially for small sample sizes. This is expected behavior for any nonparametric test, as weak alternatives are inherently difficult to detect. The proposed procedure exhibits this limitation naturally; the departure functional $T_{\beta }\left(F\right)$ is close to zero when F is near the exponential boundary, and finite-sample variability can obscure this small signal. Practitioners should therefore interpret non-rejection with caution, particularly when sample sizes are limited. The empirical MRL diagnostic recommended in Section 5.1 can provide complementary evidence in such cases.

## 5. Real Data Applications

This section illustrates the practical implementation of the proposed entropy-based scale-free L-statistic using three real datasets. The purpose is not only to report rejection decisions, but also to show how the proposed statistic can be combined with graphical mean-residual-life diagnostics and standard goodness-of-fit summaries to assess whether the exponential model is adequate or whether the data provide evidence of DMRL aging. The notation of the preceding sections is retained throughout.

### 5.1. Empirical MRL Diagnostic and Testing Procedure

For a random sample $X_{1},X_{2},\ldots ,X_{n}$ of positive observations, the empirical MRL function at a threshold t is computed as

$${\hat{m}}_{n}\left(t\right)=\frac{\sum_{i=1}^{n}\left(X_{i}-t\right)I\left(X_{i}>t\right)}{\sum_{i=1}^{n}I\left(X_{i}>t\right)},$$

whenever the denominator is non-zero. Here, $I\left(\cdot \right)$ denotes the indicator function. Equivalently, using the empirical distribution function $F_{n}$, the same quantity may be written in integral form as

$${\hat{m}}_{n}\left(t\right)=\frac{1}{1-F_{n}\left(t\right)}\int_{t}^{\infty }\left(1-F_{n}\left(x\right)\right)\;dx.$$

Under standard sampling conditions, ${\hat{m}}_{n}\left(t\right)$ is a uniformly strongly consistent estimator of the true MRL function over fixed intervals, as established by Yang [29] and further generalized by Hall and Wellner [30]. Since the DMRL property corresponds to a non-increasing MRL function, a decreasing empirical MRL curve provides useful preliminary evidence of DMRL aging. This graphical assessment is used here as a diagnostic tool before applying the proposed scale-free statistic ${\hat{T}}_{\beta }^{⋆}$.

To complement visual inspection, the applications also use two simple quantitative diagnostics when relevant: the Pearson correlation between the ordered observations and fitted exponential quantiles (rQQ), and a one-sided Kendall trend test between the threshold and the empirical MRL values. For the latter, thresholds are restricted to those leaving at least five surviving observations to reduce extreme-tail instability; a significantly negative Kendall coefficient supports a decreasing MRL pattern.

### 5.2. Application 1: Epidemic Infection Intensities

The first dataset is taken from the epidemic data analyzed by Kasilingam et al. [31]. It consists of the non-zero percentages of infected cases recorded for countries from the implementation of containment measures through 26 March 2020:

1.56, 8.51, 2.17, 0.37, 1.09, 9.84, 4.95, 3.18, 11.37, 2.81, 6.22, 1.87, 9.05, 2.44, 1.38, 4.17, 3.74, 1.37, 2.33, 7.80, 2.10, 0.47, 2.54, 4.92, 0.09, 0.18, 1.72, 1.02, 0.62, 2.34, 0.50, 2.37, 3.65, 0.59, 5.76, 2.14, 0.88, 0.95, 4.17, 2.25.

Although these observations are not conventional failure times, they are non-negative intensity-type measurements and are used here only as an illustrative dataset for assessing whether the proposed statistic refrains from falsely detecting DMRL aging when the exponential model is not strongly contradicted. Four candidate models were fitted: exponential, Weibull, log-normal, and gamma. The fitted models were compared using the maximized log-likelihood, Akaike information criterion (AIC), Bayesian information criterion (BIC), Kolmogorov-Smirnov (K-S) statistic, and the corresponding K-S *p*-value. The results are reported in Table 13.

The results in Table 13 show that the exponential model has the smallest AIC, BIC, and K-S statistic and remains a statistically plausible description of Dataset 1. However, the AIC differences relative to the gamma and Weibull models are only 0.877 and 1.021, respectively, both below the conventional threshold of 2 for substantial separation. The corresponding Akaike weights are approximately 0.427 for the exponential model, 0.275 for gamma, and 0.256 for Weibull. Thus, the exponential model is the leading parsimonious candidate, but the information criteria do not decisively exclude these alternatives; the data are compatible with more than one parametric description.

**Figure 1:**
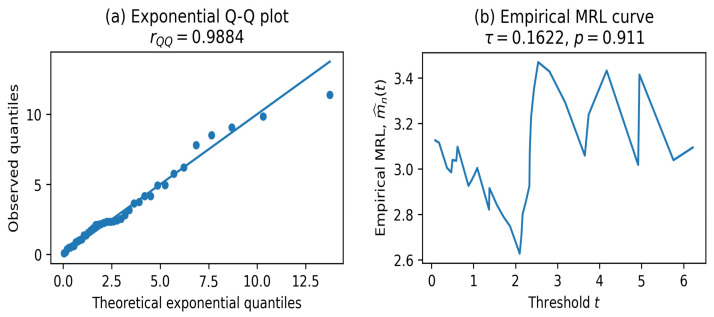
Exponential Q-Q plot and empirical MRL curve for Dataset 1.

The graphical diagnostics in Figure 1 are consistent with this qualified model-selection conclusion. The exponential Q-Q plot follows the reference line reasonably well, and the empirical MRL curve does not display a persistent decreasing trend over the main range of the data. Using the prespecified default β = 2.0, the proposed scale-free statistic is 0.02153 with an empirical *p*-value of 0.3040. Sensitivity checks at β = 1.5 and β = 4.0 lead to the same non-rejection decision. Hence, the exponential null is not rejected, which is consistent with the absence of decisive AIC separation and with the graphical diagnostics.

### 5.3. Application 2: Geoelectrical Aquifer Thickness Measurements

The second dataset, reported by Thomas and Jose [32], consists of *n* = 77 independent geoelectrical measurements of aquifer thickness in meters. These observations provide a contrasting case in which the exponential model is strongly challenged by both numerical and graphical diagnostics:

10.49, 8.80, 12.42, 4.58, 6.85, 4.58, 5.00, 4.75, 4.75, 12.25, 9.50, 13.54, 10.42, 4.65, 9.88, 6.21, 8.60, 7.06, 7.96, 7.89, 9.70, 13.90, 12.65, 10.00, 12.65, 12.07, 9.80, 13.54, 9.82, 13.54, 12.42, 12.73, 12.22, 12.25, 12.32, 8.75, 12.00, 17.50, 11.88, 13.13, 13.56, 15.44, 13.22, 7.28, 11.70, 11.70, 11.60, 10.90, 11.84, 8.00, 10.20, 5.77, 13.90, 4.58, 12.07, 15.44, 10.20, 11.00, 8.50, 10.99, 10.39, 9.90, 13.94, 15.21, 13.56, 9.00, 20.47, 15.22, 11.50, 13.90, 13.22, 10.48, 15.48, 9.80, 12.21, 13.56, 7.04.

The same four candidate distributions were fitted to these data. The resulting goodness-of-fit summaries are given in Table 14.

The results in Table 14 show that the exponential model provides a poor fit to Dataset 2, with a large K-S statistic and a K-S *p*-value close to zero. In contrast, the Weibull model gives the best overall fit among the four candidate families, with the lowest AIC and BIC values and a small K-S statistic. Under the Weibull model, the estimated shape and scale parameters are 3.7634 and 12.0098, respectively, suggesting a pronounced non-exponential pattern.

**Figure 2:**
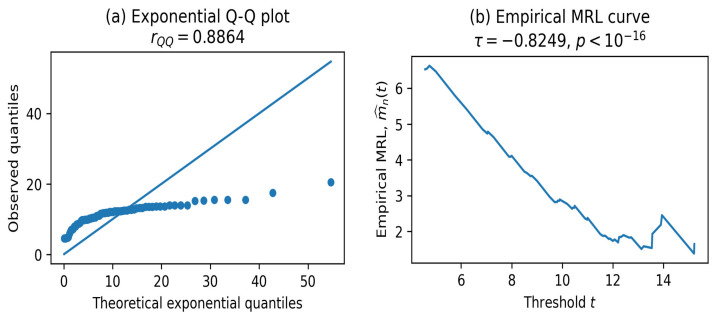
Exponential Q-Q plot and empirical MRL curve for Dataset 2.

The graphical diagnostics in Figure 2 are supported by quantitative summaries. The exponential Q-Q plot correlation coefficient is rQQ = 0.8864, substantially below the near-linear value observed for Dataset 1. For the empirical MRL curve, a one-sided Kendall trend test computed over distinct thresholds retaining at least five survivors gives τ = −0.8249 with *p* < 1 × 10^−18^, providing strong evidence of a decreasing MRL pattern. Using the prespecified default β = 2.0, the proposed scale-free statistic is 0.64378 with empirical *p*-value < 0.0001; sensitivity checks at β = 1.5 and β = 4.0 yield the same rejection. Accordingly, the exponential null is rejected in favor of a DMRL alternative. The numerical trend measures reinforce, rather than replace, the graphical interpretation and indicate that expected remaining aquifer thickness decreases systematically as the observed thickness threshold increases.

### 5.4. Application 3: Flexural Strength of Glass Material

The third dataset consists of the flexural strength of glass material (in MPa units), measured using the three-point bending method and reported by Xu et al. [33]. The dataset comprises *n* = 40 observations:

47.7, 50.2, 52.4, 52.5, 52.9, 53.8, 53.9, 54.6, 54.7, 54.9, 55.3, 55.5, 56.4, 57.5, 59.0, 60.0, 61.1, 61.4, 62.4, 62.7, 63.2, 63.5, 64.2, 65.4, 65.4, 65.6, 66.3, 66.6, 66.6, 66.8, 67.2, 67.5, 67.6, 68.0, 68.4, 69.6, 70.4, 70.7, 72.6, 74.4.

**Figure 3:**
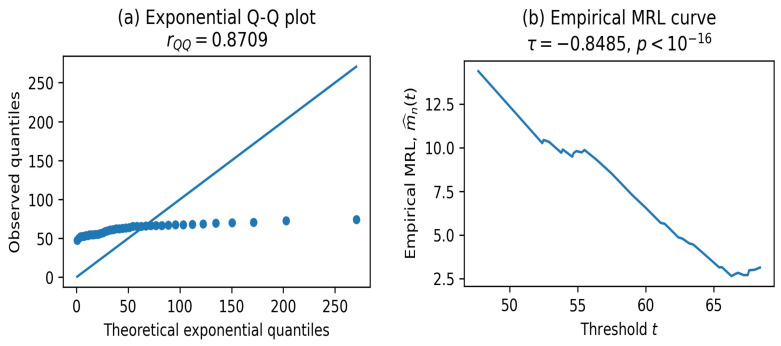
Exponential Q-Q plot and empirical MRL curve for Dataset 3.

Basu et al. [34] previously analyzed this dataset and fitted a Weibull distribution, estimating the shape and scale parameters to be 10.69151 and 64.71111, respectively. To evaluate the exponentiality assumption for this material strength data, we apply the proposed testing procedure alongside graphical diagnostics. The exponential Q-Q plot in Figure 3 reveals a systematic departure from the reference line, and the empirical MRL curve exhibits a clear decreasing trend over the main support of the data, both of which are indicative of DMRL aging behavior. Quantitatively, the exponential Q-Q correlation is rQQ = 0.8709, while the one-sided Kendall trend test gives τ = −0.8485 with *p* < 1 × 10^−16^.

Using the prespecified default β = 2.0, the proposed scale-free statistic is 0.82556 with empirical *p*-value < 0.0001. Sensitivity checks at β = 1.5 and β = 4.0 lead to the same strong rejection. Consequently, the exponential null hypothesis is rejected in favor of a DMRL alternative at the 5% significance level. From a practical reliability perspective, the glass-strength data exhibit a structured decrease in residual strength rather than memoryless exponential behavior, an important consideration for material safety assessment and engineering design.

### 5.5. Summary of the Empirical Findings

The three real-data applications illustrate diverse empirical behaviors and demonstrate the practical utility of the proposed methodology across different fields. For Dataset 1, the small AIC differences indicate that several parametric models are plausible, while the default β = 2.0 test, Q-Q plot, and empirical MRL behavior provide no strong evidence against exponentiality in the DMRL direction. For Datasets 2 and 3, the parametric comparisons, quantitative and graphical diagnostics, and the default-β test consistently indicate significant departures from exponentiality toward DMRL aging. These contrasting examples show how the proposed statistic can be used with model-selection and residual-life diagnostics to support transparent applied decisions.

## 6. Conclusions and Future Research

This paper introduced an entropy-based scale-free L-statistic for testing exponentiality against DMRL aging. The proposed procedure is motivated by a mean-residual-life characterization of the DMRL class and is formulated through a cumulative residual Tsallis entropy-type departure functional. By showing that this functional admits an L-functional representation, the method yields a nonparametric statistic expressed directly in terms of order statistics. The normalization by the sample mean removes dependence on the unknown exponential scale parameter and leads to a test that is invariant under positive scale transformations.

The distributional properties of the statistic were established under the exponential null model. In particular, the exact finite-sample null distribution was derived through normalized spacings, and asymptotic normality was obtained under standard regularity conditions. These results provide both a finite-sample calibration route and a large-sample theoretical justification for the proposed test. The Monte Carlo study under linear failure rate, gamma, and Weibull DMRL alternatives showed that the procedure maintains empirical size close to the nominal level and provides competitive power relative to established DMRL tests, especially for moderate and pronounced departures from exponentiality.

The real-data applications further demonstrated the interpretability of the proposed approach. Dataset 1 showed no significant DMRL departure under the prespecified default β = 2.0, with model-selection uncertainty explicitly acknowledged. Datasets 2 and 3 produced strong rejections supported by Q-Q and empirical-MRL diagnostics, including a quantitative decreasing-trend assessment for Dataset 2. These examples illustrate a practical workflow that avoids choosing β after observing the alternative family.

Several extensions remain promising for future research. First, the entropy-based L-statistic framework may be adapted to other aging classes, including increasing failure rate (IFR), increasing failure rate average (IFRA), new better than used (NBU), new better than used in expectation (NBUE), and their dual classes, by identifying suitable characterizing inequalities and associated departure functionals. Second, censored-data versions of the test would be valuable for right-censored, progressively censored, and interval-censored lifetime samples, which commonly arise in survival analysis and reliability experiments. Third, extensions to dependent and multivariate lifetime data may be developed for settings involving shared frailty, copula-based dependence, recurrent events, or competing risks.

Further methodological developments may also focus on computation and adaptability. Bootstrap or permutation calibration could improve finite-sample accuracy when exact null calculations are difficult or when sample sizes are small. Another important issue is the data-driven selection of the tuning parameter β, since adaptive choices may improve power while preserving size control. Finally, the proposed approach may be extended to goodness-of-fit testing for broader parametric lifetime models and to change-point, degradation, or dynamic reliability settings in which aging behavior evolves over time. These directions would broaden the role of entropy-based nonparametric methods in reliability theory, survival analysis, and lifetime-data modeling.

Several limitations of the proposed method warrant acknowledgment. First, β controls sensitivity. The revised benchmark and applications use the prespecified family-neutral default β = 2.0, but no fixed choice is uniformly optimal; data-driven adaptive selection with valid size control remains an important topic. Second, the test is designed for DMRL alternatives and may have limited power against qualitatively different departures, such as bathtub-shaped hazards. Third, exact finite-sample evaluation can be unstable when normalized-spacing coefficients are nearly equal or repeated, in which case null simulation is preferable. Fourth, the present method assumes complete uncensored data. Fifth, weak DMRL departures may require substantial samples. The test should therefore be interpreted jointly with Q-Q plots, empirical MRL diagnostics, and standard goodness-of-fit summaries.

## Figures and Tables

**Table 1:** Comparative positioning of representative exponentiality tests relevant to DMRL aging.

Approach/References	Main Construction	Principal Strength	Position Relative to the Proposed Method
Classical aging-inequality and TTT procedures [2,3,4,5]	Aging inequalities and total-time-on-test transforms	Established nonparametric tools with a direct reliability motivation	They are not derived from an entropy functional and do not provide the same unified scale-free L-functional framework with both exact and asymptotic null theory.
MRL-based procedures [6,7,8,9]	Contrasts based on the monotonicity or structure of the mean residual life function	Direct interpretation in terms of lifetime aging	They do not combine a CRTE-type departure measure, an order-statistic L-representation, and an exact normalized-spacing null distribution.
Quantile-, dispersion-, and moment-based procedures [10,11,12,13,14,15,16,17]	Distributional inequalities, residual-life dispersion, quantiles, or moment contrasts	Flexible nonparametric detection of aging departures	The interpretation and null theory are procedure-specific rather than integrated in a single information-theoretic, scale-free construction.
Recent entropy-based procedures [18,20]	Cumulative residual Rényi or Tsallis entropy-type departures	Information-theoretic measurement of departure from exponentiality	They do not jointly develop the DMRL characterization, scale-free order-statistic L-form, exact normalized-spacing distribution, and asymptotic theory used here.
General L-statistic exponentiality tests [19]	Linear combinations of order statistics	Computationally tractable L-statistic methodology	They are not specifically derived from a DMRL-oriented cumulative residual entropy characterization.
Recent MRL and U-statistic procedures [21,22]	MRL discrepancies or U-statistic constructions	Contemporary nonparametric exponentiality testing	They do not provide the same combined entropy-based aging interpretation, scale invariance, and exact finite-sample representation.
Proposed method	CRTE-type DMRL departure functional and a score-weighted order-statistic L-statistic	Direct DMRL interpretation, scale invariance, and a tunable weighting mechanism through β	It unifies aging interpretation, entropy-based departure measurement, exact finite-sample null theory via normalized spacings, and asymptotic normality.

**Table 2:** Summary of notation and abbreviations.

Symbol	Description
*X*	Non-negative lifetime random variable
*F*(*x*), $\bar{F}\left(t\right),$*f*(*x*)	Cumulative distribution function, survival function, and density function of X
*m*(*t*)	Mean residual life (MRL) function
*μ* = *E*[*X*]	Mean of the lifetime distribution
*X*_(1)_ ≤ … ≤ *X*_(*n*)_	Order statistics of a random sample of size *n*
$\bar{X}$	Sample mean
*S_β_*(*X*)	Cumulative residual Tsallis entropy of order β
*J_β_*(*u*)	Score function for the L-functional representation
*Q*(*u*) *= F*^−1^(*u*)	Quantile function
*T_β_*(*F*)	Entropy-based population departure functional for DMRL aging
$T_{\beta }^{⋆}(F)$	Population scale-free departure functional
${\hat{T}}_{\beta }$	Empirical L-statistic
${{\hat{T}}^{⋆}}_{\beta }$	Scale-free sample test statistic
*Y_j_*	Normalized spacing
*c_j,n_* ^(^ *^β^* ^)^	Deterministic coefficient in the exact null distribution
$\sigma_{\beta^{2}}(F)$	Asymptotic variance of the empirical L-statistic
*H*_0_, *H*_1_	Null hypothesis (exponentiality) and alternative hypothesis (strictly DMRL)
$z_{1-\alpha }$	Standard normal quantile
*α*	Significance level of the test
*Γ*(·)	Gamma function
*I*(·)	Indicator function
*n*	Sample size
*β*	Entropy order and tuning parameter of the proposed statistic
*λ*	Rate parameter of the exponential null distribution
*θ*	Shape or departure parameter used for the alternative lifetime models
*B*	Number of Monte Carlo replications
MRL	Mean residual life
DMRL	Decreasing mean residual life
IMRL	Increasing mean residual life
CRTE	Cumulative residual Tsallis entropy
LFR	Linear failure rate
IFR	Increasing failure rate
IFRA	Increasing failure rate average
NBU	New better than used
NBUE	New better than used in expectation
AIC	Akaike information criterion
BIC	Bayesian information criterion
K-S	Kolmogorov-Smirnov goodness-of-fit statistic
Q-Q	Quantile-quantile plot

**Table 3:** Alternative distributions used in the simulation study.

Distribution	Survival Function	Support
LFR	${\bar{F}}_{1}\left(x\right)=exp\left\{-\left(x+\frac{\theta x^{2}}{2}\right)\right\}$	x>0, θ>0
Gamma	${\bar{F}}_{2}\left(x\right)=\frac{1}{\Gamma \left(\theta \right)}\int_{x}^{\infty }t^{\theta -1}e^{-t}\;dt$	x>0, θ>0
Weibull	${\bar{F}}_{3}\left(x\right)=exp\left(-x^{\theta }\right)$	x>0, θ>0

**Table 4:** Empirical power of the proposed test under the LFR alternative at significance level 0.05.

Sample Size	θ	$T_{\beta =1.5}^{⋆}$	$T_{\beta =2.0}^{⋆}$	$T_{\beta =2.5}^{⋆}$	$T_{\beta =3.0}^{⋆}$	$T_{\beta =3.5}^{⋆}$	$T_{\beta =4.0}^{⋆}$	$T_{\beta =4.5}^{⋆}$	$T_{\beta =5.0}^{⋆}$
20	0.25	0.1210	0.1192	0.1272	0.0972	0.1102	0.1158	0.0950	0.1204
	0.75	0.2288	0.2436	0.2428	0.2112	0.2144	0.2232	0.1910	0.2048
	1.25	0.2868	0.3116	0.3274	0.2940	0.2856	0.2746	0.2800	0.2546
	1.75	0.4116	0.4208	0.3820	0.3654	0.3536	0.3172	0.3218	0.3378
	2.25	0.4704	0.4726	0.4198	0.4312	0.3966	0.3968	0.3842	0.3580
30	0.25	0.1448	0.1350	0.1370	0.1464	0.1318	0.1306	0.1054	0.1086
	0.75	0.3318	0.3378	0.3234	0.3022	0.2800	0.2762	0.2552	0.2654
	1.25	0.4990	0.4674	0.4386	0.4018	0.4222	0.3906	0.3596	0.3638
	1.75	0.5688	0.5340	0.5290	0.4980	0.4790	0.4710	0.4700	0.4270
	2.25	0.6350	0.6102	0.6206	0.5646	0.5562	0.5416	0.4998	0.5044
40	0.25	0.1956	0.1630	0.1778	0.1602	0.1614	0.1608	0.1254	0.1526
	0.75	0.4400	0.4208	0.4210	0.3698	0.3852	0.3282	0.3210	0.3094
	1.25	0.5796	0.5890	0.5388	0.5282	0.5164	0.4768	0.4550	0.4296
	1.75	0.6966	0.6726	0.6644	0.6500	0.5836	0.5590	0.5444	0.5302
	2.25	0.7802	0.7646	0.7158	0.6828	0.6536	0.6684	0.6020	0.6068
50	0.25	0.2088	0.2284	0.1864	0.2082	0.2066	0.1638	0.1536	0.1460
	0.75	0.5218	0.4894	0.4582	0.4286	0.4162	0.4016	0.3908	0.3792
	1.25	0.6862	0.6734	0.6410	0.6056	0.5774	0.5670	0.5152	0.5118
	1.75	0.7806	0.7742	0.7550	0.7272	0.6854	0.6558	0.6442	0.6180
	2.25	0.8638	0.8208	0.8126	0.7878	0.7632	0.7440	0.7008	0.6912

**Table 5:** Empirical power of the proposed test under the Gamma alternative at significance level 0.05.

Sample Size	θ	$T_{\beta =1.5}^{⋆}$	$T_{\beta =2.0}^{⋆}$	$T_{\beta =2.5}^{⋆}$	$T_{\beta =3.0}^{⋆}$	$T_{\beta =3.5}^{⋆}$	$T_{\beta =4.0}^{⋆}$	$T_{\beta =4.5}^{⋆}$	$T_{\beta =5.0}^{⋆}$
20	1.2	0.1132	0.1130	0.1172	0.1296	0.1380	0.1262	0.1192	0.1132
	1.6	0.3284	0.3620	0.3614	0.4202	0.3848	0.3952	0.4280	0.3990
	2.0	0.5598	0.6078	0.6706	0.6570	0.7032	0.6648	0.6678	0.6568
	2.2	0.6564	0.7200	0.7662	0.7754	0.7378	0.7840	0.7926	0.7950
	2.6	0.8066	0.8790	0.8920	0.8976	0.9160	0.9186	0.9126	0.9252
30	1.2	0.1620	0.1498	0.1592	0.1554	0.1708	0.1632	0.1624	0.1448
	1.6	0.4260	0.4692	0.5366	0.5318	0.5388	0.5526	0.5490	0.5400
	2.0	0.7634	0.7968	0.8224	0.8344	0.8416	0.8434	0.8514	0.8460
	2.2	0.8300	0.8908	0.9110	0.9310	0.9212	0.9334	0.9180	0.9318
	2.6	0.9428	0.9736	0.9818	0.9844	0.9866	0.9832	0.9866	0.9864
40	1.2	0.1582	0.1708	0.1806	0.1848	0.2104	0.1852	0.2082	0.1960
	1.6	0.5474	0.5996	0.6286	0.6564	0.6598	0.6814	0.6782	0.6686
	2.0	0.8558	0.8870	0.9156	0.9296	0.9278	0.9428	0.9484	0.9444
	2.2	0.9146	0.9616	0.9682	0.9726	0.9842	0.9760	0.9800	0.9810
	2.6	0.9838	0.9950	0.9974	0.9988	0.9984	0.9986	0.9986	0.9984
50	1.2	0.1746	0.2052	0.2200	0.2112	0.2334	0.2258	0.2412	0.2182
	1.6	0.6312	0.6986	0.7292	0.7528	0.7706	0.7712	0.7994	0.7724
	2.0	0.9198	0.9502	0.9566	0.9734	0.9732	0.9792	0.9780	0.9784
	2.2	0.9672	0.9830	0.9902	0.9888	0.9940	0.9954	0.9944	0.9938
	2.6	0.9956	0.9982	1.0000	0.9998	0.9998	0.9998	1.0000	1.0000

**Table 6:** Empirical power of the proposed test under the Weibull alternative at significance level 0.05.

Sample Size	θ	$T_{\beta =1.5}^{⋆}$	$T_{\beta =2.0}^{⋆}$	$T_{\beta =2.5}^{⋆}$	$T_{\beta =3.0}^{⋆}$	$T_{\beta =3.5}^{⋆}$	$T_{\beta =4.0}^{⋆}$	$T_{\beta =4.5}^{⋆}$	$T_{\beta =5.0}^{⋆}$
20	1.1	0.1080	0.1174	0.1134	0.1168	0.1084	0.1170	0.1172	0.1094
	1.3	0.3262	0.3326	0.3658	0.3448	0.3444	0.3428	0.3336	0.3418
	1.5	0.6010	0.6414	0.6402	0.6522	0.6504	0.6582	0.6178	0.6428
	1.7	0.8234	0.8388	0.8774	0.8586	0.8604	0.8440	0.8254	0.8372
	1.9	0.9546	0.9672	0.9632	0.9610	0.9528	0.9618	0.9530	0.9426
30	1.1	0.1322	0.1368	0.1422	0.1152	0.1582	0.1462	0.1510	0.1480
	1.3	0.4476	0.4884	0.5118	0.4916	0.4630	0.4816	0.4990	0.4720
	1.5	0.8280	0.8072	0.8478	0.8370	0.8228	0.8148	0.8010	0.7982
	1.7	0.9518	0.9652	0.9656	0.9590	0.9650	0.9604	0.9532	0.9572
	1.9	0.9948	0.9962	0.9962	0.9972	0.9968	0.9948	0.9924	0.9920
40	1.1	0.1736	0.1544	0.1678	0.1514	0.1738	0.1730	0.1654	0.1828
	1.3	0.5764	0.5814	0.6182	0.6056	0.6080	0.5990	0.6032	0.5874
	1.5	0.9074	0.9146	0.9150	0.9274	0.9180	0.9212	0.9202	0.9104
	1.7	0.9928	0.9940	0.9952	0.9948	0.9924	0.9916	0.9874	0.9898
	1.9	0.9994	1.0000	0.9998	0.9996	0.9994	1.0000	0.9994	0.9994
50	1.1	0.1772	0.1600	0.2020	0.1720	0.1752	0.1978	0.2036	0.1908
	1.3	0.6624	0.6962	0.7058	0.6928	0.7130	0.7014	0.6846	0.6874
	1.5	0.9566	0.9710	0.9696	0.9676	0.9674	0.9598	0.9584	0.9576
	1.7	0.9988	0.9988	0.9982	0.9982	0.9990	0.9990	0.9974	0.9982
	1.9	1.0000	1.0000	1.0000	1.0000	1.0000	1.0000	1.0000	1.0000

**Table 7:** Benchmark tests for exponentiality against DMRL alternatives.

Test	Reference	Notation
1	Hollander and Proschan [2]	*S*_1_ = *V*/$\bar{X}$
2	Abouammoh and Khalique [3]	*S*_2_ = *M*_1_
3	Aly [6]	*S*_3_ = *γ*($F_{n}$)/$\bar{X}$
4	Fernandez-Ponce et al. [10]	*S*_4_ = Δ($F_{n}$)
5	Belzunce et al. [12]	*S*_5_ = $\Delta^{⋆}$*_DMRL_*(*n*)
6	Abu-Youssef [13]	*S*_6_ = $\Delta_{n}$
7	Ahmad and Mugdadi [14]	*S*_7_ = *δ*(3)
8	Li et al. [15]	*S*_8_ = $U_{n}$/$\bar{X}$
9	Anis [17]	*S*_9_ = $\delta_{n}$/$\bar{X}$
10	Lorenzo et al. [16]	*S*_10_ = *K*/$\bar{X}$
11	Zardasht [18]	$S_{11}(\alpha )$= $R_{n}$(*α*)

**Table 8:** Empirical Type I error rates of the benchmark tests and the proposed statistic at significance level 0.05.

*n*	S1	S2	S3	S4	S5	S6	S7	S8	S9	S10	$S_{11}(\alpha =2)$	$T_{\beta =2.0}^{⋆}$
5	0.0598	0.0538	0.0536	0.0502	0.0559	0.0575	0.0526	0.0525	0.0502	0.0564	0.0525	0.0524
10	0.0544	0.0531	0.0523	0.0511	0.0548	0.0540	0.0501	0.0544	0.0491	0.0587	0.0511	0.0498
15	0.0512	0.0486	0.0478	0.0528	0.0512	0.0517	0.0463	0.0505	0.0499	0.0449	0.0480	0.0468
20	0.0457	0.0463	0.0469	0.0498	0.0490	0.0490	0.0486	0.0475	0.0475	0.0472	0.0471	0.0472
30	0.0510	0.0523	0.0522	0.0475	0.0531	0.0484	0.0524	0.0491	0.0488	0.0497	0.0536	0.0530
50	0.0482	0.0542	0.0518	0.0538	0.0491	0.0478	0.0535	0.0489	0.0459	0.0502	0.0580	0.0512
100	0.0460	0.0497	0.0487	0.0482	0.0487	0.0470	0.0469	0.0477	0.0462	0.0515	0.0507	0.0528

**Table 9:** Power comparison under the LFR alternative at significance level 0.05.

θ	*n*	S1	S2	S3	S4	S5	S6	S7	S8	S9	S10	$S_{11}(\alpha =2)$	$T_{\beta =2.0}^{⋆}$
0.25	5	0.0732	0.0728	0.0713	0.0638	0.0723	0.0717	0.0717	0.0634	0.0422	0.0724	0.0733	**0.0788**
	10	0.0811	0.0873	0.0876	0.0717	0.0835	0.0809	0.0842	0.0740	0.0514	0.0894	0.0860	**0.0968**
	15	0.0900	0.0964	0.0956	0.0867	0.0983	0.0895	0.0964	0.0838	0.0640	0.0889	**0.0991**	0.0966
	20	0.1003	0.1102	0.1098	0.1010	0.1110	0.0999	0.1080	0.0941	0.0784	0.1094	0.1076	**0.1222**
	30	0.1334	**0.1547**	0.1527	0.1155	0.1481	0.1291	0.1499	0.1231	0.0974	0.1361	0.1514	0.1530
	50	0.1872	0.2147	0.2071	0.1727	0.2135	0.1739	0.2120	0.1759	0.1325	0.1813	0.2186	**0.2192**
	100	0.3198	0.3499	0.3561	0.2858	0.3638	0.2942	0.3413	0.3044	0.2381	0.2983	**0.3707**	0.3556
0.75	5	0.0968	0.0983	0.0973	0.0788	0.1004	0.0901	0.0958	0.0697	0.0320	0.0985	0.0999	**0.1058**
	10	0.1216	0.1443	0.1424	0.1030	0.1414	0.1175	0.1373	0.0954	0.0530	0.1367	**0.1463**	0.1454
	15	0.1502	0.1842	0.1810	0.1397	0.1821	0.1436	0.1759	0.1249	0.0756	0.1619	**0.1850**	0.1784
	20	0.1845	0.2255	0.2227	0.1713	0.2241	0.1823	0.2204	0.1536	0.1086	0.2010	0.2217	**0.2258**
	30	0.2656	0.3375	0.3251	0.2235	0.3261	0.2551	0.3287	0.2293	0.1602	0.2837	**0.3377**	0.3354
	50	0.4169	0.5113	0.4982	0.3618	0.5086	0.3927	0.5041	0.3707	0.2628	0.4216	**0.5271**	0.5026
	100	0.7098	0.7832	0.7869	0.6188	0.7972	0.6825	0.7741	0.6676	0.5343	0.6956	**0.8108**	0.7942
1.25	5	0.1107	0.1162	0.1174	0.0872	**0.1191**	0.1041	0.1155	0.0720	0.0264	0.1145	0.1169	0.1182
	10	0.1510	0.1882	0.1872	0.1236	0.1775	0.1404	0.1750	0.1085	0.0528	0.1719	0.1886	**0.2072**
	15	0.1955	0.2521	0.2460	0.1693	0.2448	0.1860	0.2405	0.1492	0.0817	0.2144	**0.2567**	0.2354
	20	0.2410	0.3131	0.3062	0.2142	0.3076	0.2397	0.3019	0.1893	0.1295	0.2686	0.3134	**0.3386**
	30	0.3578	0.4699	0.4569	0.2892	0.4584	0.3463	0.4553	0.2925	0.2026	0.3877	**0.4754**	0.4674
	50	0.5554	0.6817	0.6693	0.4682	0.6785	0.5292	0.6763	0.4865	0.3558	0.5784	**0.6935**	0.6804
	100	0.8621	0.9235	0.9265	0.7468	0.9331	0.8467	0.9192	0.8250	0.6913	0.8611	**0.9416**	0.9334

Note: Bold values indicate the maximum empirical power among the compared procedures for each configuration.

**Table 10:** Power comparison under the Gamma alternative at significance level 0.05.

θ	*n*	S1	S2	S3	S4	S5	S6	S7	S8	S9	S10	$S_{11}(\alpha =2)$	$T_{\beta =2.0}^{⋆}$
1.2	5	0.0763	0.0767	0.0758	0.0604	0.0784	0.0716	0.0716	0.0537	0.0279	0.0768	0.0747	0.0848
	10	0.0709	0.0948	0.0932	0.0682	0.0867	0.0662	0.0894	0.0556	0.0317	0.0913	0.0954	0.0956
	15	0.0753	0.1062	0.1026	0.0767	0.0957	0.0742	0.1024	0.0621	0.0368	0.0908	**0.1114**	0.0930
	20	0.0762	0.1166	0.1127	0.0786	0.1040	0.0746	0.1160	0.0619	0.0468	0.1025	**0.1254**	0.1200
	30	0.0874	0.1561	0.1434	0.0853	0.1240	0.0822	0.1511	0.0720	0.0496	0.1221	**0.1730**	0.1528
	50	0.0969	0.2101	0.1810	0.1125	0.1530	0.0891	0.2081	0.0811	0.0525	0.1640	**0.2384**	0.2032
	100	0.1380	0.3329	0.2682	0.1583	0.2355	0.1235	0.3263	0.1192	0.0788	0.2625	**0.3617**	0.3378
1.6	5	0.0995	0.1239	0.1208	0.0715	0.1142	0.0831	0.1082	0.0407	0.0074	0.1128	0.1222	0.1336
	10	0.1215	0.2123	0.2046	0.1052	0.1796	0.1132	0.2000	0.0630	0.0152	0.1656	**0.2215**	0.2180
	15	0.1283	0.2988	0.2796	0.1163	0.2199	0.1271	0.2858	0.0711	0.0274	0.2208	**0.3246**	0.2630
	20	0.1346	0.3398	0.3130	0.1392	0.2538	0.1458	0.3285	0.0806	0.0435	0.2701	**0.3764**	0.3656
	30	0.1700	0.4944	0.4333	0.1712	0.3436	0.1878	0.4843	0.1142	0.0671	0.3842	**0.5496**	0.4810
	50	0.2283	0.7126	0.6151	0.2539	0.5116	0.2511	0.7005	0.1598	0.1038	0.5800	**0.7699**	0.7076
	100	0.3924	0.9345	0.8535	0.4240	0.7791	0.4259	0.9280	0.2961	0.1949	0.8681	**0.9605**	0.9434
2.0	5	0.1326	0.1716	0.1719	0.0818	0.1591	0.1075	0.1612	0.0329	0.0026	0.1437	0.1720	**0.1888**
	10	0.1481	0.3290	0.3136	0.1202	0.2578	0.1452	0.3052	0.0497	0.0075	0.2538	0.3479	**0.3642**
	15	0.1802	0.4938	0.4601	0.1505	0.3664	0.2035	0.4828	0.0671	0.0276	0.3860	**0.5320**	0.4698
	20	0.1898	0.6003	0.5500	0.1888	0.4392	0.2448	0.5785	0.0850	0.0532	0.4887	**0.6512**	0.6126
	30	0.2540	0.8033	0.7329	0.2428	0.5983	0.3359	0.7875	0.1317	0.0953	0.6726	**0.8524**	0.7978
	50	0.3620	0.9523	0.8972	0.3838	0.8150	0.4838	0.9470	0.2208	0.1823	0.8875	**0.9742**	0.9538
	100	0.6178	0.9992	0.9922	0.6170	0.9803	0.7740	0.9991	0.4611	0.3971	0.9951	**0.9998**	0.9994

Note: Bold values indicate the maximum empirical power among the compared procedures for each configuration.

**Table 11:** Power comparison under the Weibull alternative at significance level 0.05.

θ	*n*	S1	S2	S3	S4	S5	S6	S7	S8	S9	S10	$S_{11}(\alpha =2)$	$T_{\beta =2.0}^{⋆}$
1.1	5	0.0677	0.0729	0.0706	0.0593	0.0704	0.0620	0.0718	0.0491	0.0317	0.0687	0.0707	**0.0732**
	10	0.0683	0.0844	0.0840	0.0665	0.0763	0.0667	0.0811	0.0577	0.0372	0.0855	0.0861	**0.0902**
	15	0.0733	0.1002	0.0980	0.0771	0.0903	0.0712	0.0992	0.0630	0.0446	0.0884	**0.1014**	0.0914
	20	0.0772	0.1084	0.1016	0.0822	0.0961	0.0787	0.1053	0.0678	0.0544	0.0985	0.1132	**0.1180**
	30	0.0936	0.1479	0.1363	0.0917	0.1235	0.0903	0.1432	0.0813	0.0625	0.1226	**0.1569**	0.1334
	50	0.1144	0.1968	0.1756	0.1304	0.1592	0.1051	0.1895	0.1010	0.0705	0.1563	**0.2087**	0.1818
	100	0.1600	0.2904	0.2541	0.1739	0.2315	0.1441	0.2841	0.1436	0.1007	0.2344	0.2976	**0.3042**
1.3	5	0.1024	0.1197	0.1173	0.0759	0.1154	0.0925	0.1188	0.0498	0.0120	0.1146	0.1222	**0.1380**
	10	0.1196	0.1942	0.1912	0.1049	0.1643	0.1114	0.1851	0.0703	0.0232	0.1751	0.2015	**0.2140**
	15	0.1437	0.2711	0.2551	0.1383	0.2237	0.1414	0.2551	0.0904	0.0438	0.2134	**0.2770**	0.2568
	20	0.1640	0.3356	0.3126	0.1620	0.2727	0.1710	0.3245	0.1102	0.0670	0.2723	**0.3467**	0.3438
	30	0.2329	0.4897	0.4453	0.2110	0.3881	0.2395	0.4764	0.1640	0.1025	0.3776	**0.5104**	0.4904
	50	0.3408	0.7024	0.6359	0.3425	0.5797	0.3478	0.6918	0.2589	0.1708	0.5658	**0.7239**	0.7002
	100	0.5940	0.9382	0.8932	0.5695	0.8610	0.5962	0.9336	0.5006	0.3513	0.8506	**0.9413**	0.9372
1.5	5	0.1462	0.1835	0.1800	0.0919	0.1682	0.1207	0.1738	0.0468	0.0053	0.1682	0.1857	**0.1944**
	10	0.1828	0.3509	0.3405	0.1433	0.2829	0.1847	0.3272	0.0772	0.0177	0.2966	0.3566	**0.3778**
	15	0.2289	0.5034	0.4790	0.2021	0.4127	0.2530	0.4758	0.1082	0.0503	0.3846	**0.5113**	0.4846
	20	0.2809	0.6226	0.5805	0.2447	0.5135	0.3275	0.6002	0.1492	0.0947	0.5078	0.6389	**0.6428**
	30	0.4048	0.8245	0.7744	0.3521	0.7079	0.4703	0.8124	0.2514	0.1855	0.6889	**0.8378**	0.8292
	50	0.6066	0.9700	0.9440	0.5423	0.9107	0.6913	0.9646	0.4444	0.3564	0.8975	**0.9727**	0.9676
	100	0.8919	0.9996	0.9987	0.8072	0.9973	0.9443	0.9995	0.8027	0.7273	0.9973	0.9998	**1.0000**

Note: Bold values indicate the maximum empirical power among the compared procedures for each configuration.

**Table 12:** Average power ranks across the 63 configurations in Table 9, Table 10 and Table 11 (smaller ranks are better).

Procedure	LFR	Gamma	Weibull	Overall
S11	2.24	1.52	1.57	1.78
Proposed T* (beta = 2.0)	2.43	2.52	2.14	2.37
S2	3.21	2.67	2.62	2.83
S3	4.67	4.71	4.69	4.69
S7	5.71	4.40	4.12	4.75
S5	3.67	6.33	6.29	5.43
S10	6.76	6.00	6.57	6.44
S1	7.52	8.71	8.76	8.33
S6	9.02	8.88	8.71	8.87
S4	10.38	9.24	9.52	9.71
S8	10.38	11.00	11.00	10.79
S9	12.00	12.00	12.00	12.00

**Table 13:** Parametric model selection criteria for Dataset 1.

Distribution	Log-Likelihood	AIC	BIC	K-S	*p*-Value
Exponential	−85.730	173.461	175.150	0.087	0.916
Weibull	−85.241	174.482	177.859	0.098	0.829
Log-normal	−87.069	178.137	181.515	0.119	0.621
Gamma	−85.169	174.338	177.715	0.093	0.876

**Table 14:** Parametric model selection criteria for Dataset 2.

Distribution	Log-Likelihood	AIC	BIC	K-S	*p*-Value
Exponential	−260.615	523.230	525.573	0.351	0.000
Weibull	−198.963	401.926	406.613	0.082	0.671
Log-normal	−205.928	415.857	420.544	0.144	0.082
Gamma	−202.464	408.929	413.617	0.121	0.213

## Data Availability

The data analyzed in this study, together with the R code used to implement the proposed procedure and reproduce the numerical results, are provided in Supplementary File. The original sources of the three real datasets are cited in References [31,32,33].

## Supplementary Materials

The following supporting information can be downloaded at: https://www.mdpi.com/article/10.3390/e28070755/s1. Supplementary File: Detailed proofs of Theorems 1 and 2, additional details for the L-functional representation, R code for the proposed scale-free statistic, null calibration, sensitivity simulations, and real-data applications, together with the datasets used in the applications.
