# Supplementary material for: An Entropy-Based Scale-Free L-Statistic for Exponentiality Testing in Lifetime Data with DMRL Aging
Source: Entropy (Basel). 2026 Jul 1;28(7):755. doi: 10.3390/e28070755 (PMC13408412; doi:10.3390/e28070755)
Supplement: Supplementary file 1 [file entropy-28-00755-s001.zip › entropy-4394329-supplementary.pdf]

Supplementary File

An Entropy-Based Scale-Free L-Statistic for Exponentiality Testing in Lifetime Data with DMRL Aging

Supplementary File. This file contains detailed proofs of Theorems 1 and 2, additional details supporting the L-functional representation, and self-contained R code for the proposed scale-free statistic, null calibration, sensitivity analyses, graphical diagnostics, and the three real-data applications.

S1. Mathematical Proofs

S1.1. Proof of Theorem 1

Assumptions. Let F be absolutely continuous on $[0,\infty)$, with finite mean μ and mean residual life $m(t)$. Let $\beta>0$, $\beta\neq 1$, and assume that all quantities below are finite. For the strict conclusion, assume that $m(t)<\mu$ on a set having positive probability under the proportional-hazards transform with density $f\beta(t)=\beta f(t)\bar{F}(t)$^$(\beta-1)$.

From Equation (1) in the manuscript and the definition of the departure functional in Equation (2),

$$E\big[m\big(X_\beta\big)\big] = \frac{\beta}{\beta-1}\{\mu - \int_0^\infty \overline{F}\,(x)^\beta\,dx\}.$$

$$T_\beta(F) = \frac{\beta}{\beta-1}\int_0^\infty \overline{F}\,(x)^\beta\,dx - \frac{\mu}{\beta-1}.$$

Eliminating the common integral gives the equivalent representation

$$T_\beta(F) = \mu - E\big[m\big(X_\beta\big)\big] = \int_0^\infty [\mu - m(t)]\, f_\beta(t)\,dt. \qquad \text{(S1)}$$

If F is DMRL, then $m(t)\leq m(0)=\mu$ for every $t\geq 0$. Hence the integrand in (S1) is non-negative and $T\beta(F)\geq 0$. Under the strict DMRL alternative, excluding the exponential boundary, $\mu-m(t)$ is strictly positive on a set of positive $f\beta$-measure. Therefore the integral in (S1) is strictly positive, and

$$T_\beta(F) > 0.$$

Since $\mu>0$, division by μ also yields $T_\beta^*(F)= T_\beta(F)/\mu>0$. This proves Theorem 1.

S1.2. Proof of Theorem 2

Step 1: normalized spacings. Under H_0, let $X_{(1)}<\cdots<X_{(n)}$ be the order statistics of an exponential sample with rate λ, and define $Y_j=(n-j+1)[X_{(j)}-X_{(j-1)}]$, with $X_{(0)}=0$. The variables $Y_1,\ldots,Y_n$ are independent and identically distributed exponential random variables with rate λ.

Using X(i)=Σ_{j=1}^i Yj/(n−j+1) in the empirical L-statistic and interchanging the two finite sums gives

$$\hat{T}^{\star}_{\beta,n} = \frac{\sum_{j=1}^{n} c_{j,n}(\beta) Y_j}{\sum_{j=1}^{n} Y_j}.$$

$$c_{j,n}(\beta) = \frac{1}{n-j+1} \sum_{i=j}^{n} J_\beta\left(\frac{i}{n}\right). \tag{S2}$$

Step 2: Dirichlet reduction. Set S=ΣYj and Wj=Yj/S. Because independent exponential variables are gamma variables with a common rate and unit shape, the normalized vector W=(W1,…,Wn) has the Dirichlet(1,…,1) distribution and is independent of S. Consequently,

$$\hat{T}^{\star}_{\beta,n} = \sum_{j=1}^{n} c_{j,n}(\beta) W_j. \tag{S3}$$

The Dirichlet(1,…,1) distribution is uniform on the simplex Δn−1={w:wj≥0, Σwj=1}, with constant density (n−1)!.

Step 3: distribution of the random convex combination. Assume that the knots c1,n(β),…,cn,n(β) are pairwise distinct. The volume of the truncated simplex {w∈Δn−1:Σcj,n(β)wj≤x} is the standard simplex-spline volume

$$\mathrm{Vol}\left\{w \in \Delta_{n-1}: \sum_{j=1}^{n} c_{j,n}(\beta) w_j \le x\right\} = \frac{1}{(n-1)!} \sum_{j=1}^{n} \frac{\max\{x - c_{j,n}(\beta), 0\}^{n-1}}{\prod_{k \neq j} [c_{k,n}(\beta) - c_{j,n}(\beta)]}.$$

where $(a)_+$=max(a,0). Multiplying this volume by the Dirichlet density (n−1)! yields

$$P\left(\hat{T}^{\star}_{\beta,n} \le x\right) = \sum_{j=1}^{n} \frac{\max\{x - c_{j,n}(\beta), 0\}^{n-1}}{\prod_{k \neq j} [c_{k,n}(\beta) - c_{j,n}(\beta)]}. \tag{S4}$$

For x below the smallest knot, every positive-part term in (S4) is zero. For x above the largest knot, the divided-difference identity makes the sum equal to one. Hence the support is [minj cj,n(β), maxj cj,n(β)]. Formula (S4) is exactly Equation (7) in the manuscript. If two or more knots coincide, the distribution is obtained by continuity (equivalently, by the repeated-knot B-spline limit); in computation, direct null simulation is preferable. This completes the proof.

S1.3. Additional Details for the L-Functional Representation

The integration-by-parts step used in Section 2.2 may be written explicitly as follows. Because dF(x)=f(x)dx and d[F̄(x)^β]=−βf(x)F̄(x)^(β−1)dx,

$$\int_0^\infty \bar{F}(x)^\beta \, dx = \beta \int_0^\infty x f(x) \bar{F}(x)^{\beta-1} \, dx.$$

provided the boundary term xF̄(x)^β vanishes at infinity. Combining this identity with Equation (2) and writing the resulting integral with respect to dF(x) gives

$$T_\beta(F) = \int_0^\infty x J_\beta\big(F(x)\big) \, dF(x).$$

$$J_\beta(u) = \frac{\beta^2 (1-u)^{\beta-1} - 1}{\beta - 1}, \qquad 0 < u < 1.$$

The quantile form follows from the change of variable u=F(x):

$$T_\beta(F) = \int_0^1 Q\,(u) J_\beta(u)\,du.$$

S2. Computational Reproducibility

The following code is written in base R, except for the optional parametric model-fitting routine, which uses the recommended package MASS. The simulations in the manuscript use B=5000 Monte Carlo replications and nominal level α=0.05. A fixed seed is included for reproducibility; changing the seed produces Monte Carlo-equivalent results.

- R version 4.3 or later is recommended.
- The exponential null distribution is simulated with rate 1 because the statistic is scale invariant.
- For exact replication of printed Monte Carlo entries, use the same seed and replication count used in the final master script.

S3. R Code

S3.1. Core Score Function and Scale-Free Statistic

```
## ------------------------------------------------------------
## Core statistic
## ------------------------------------------------------------

score_beta <- function(u, beta) {
  if (!is.numeric(beta) || length(beta) != 1L || beta <= 0 ||
      abs(beta - 1) < .Machine$double.eps^0.5) {
    stop("beta must be positive and different from 1.")
  }
  (beta^2 * (1 - u)^(beta - 1) - 1) / (beta - 1)
}

T_beta_star <- function(x, beta) {
  x <- as.numeric(x)
  if (length(x) < 2L || any(!is.finite(x)) || any(x < 0)) {
    stop("x must contain at least two finite non-negative observations.")
  }
  xbar <- mean(x)
  if (xbar <= 0) stop("The sample mean must be positive.")

  x_ord <- sort(x)
  n <- length(x_ord)
  weights <- score_beta(seq_len(n) / n, beta)
  mean(weights * x_ord) / xbar
```

```
}
```

S3.2. Random-Number Generators for the Alternatives

```
## ------------------------------------------------------------
## Alternative models used in Table 3
## ------------------------------------------------------------

r_lfr <- function(n, theta) {
  if (theta < 0) stop("theta must be non-negative.")
  e <- rexp(n, rate = 1)
  if (theta == 0) return(e)
  (-1 + sqrt(1 + 2 * theta * e)) / theta
}

r_gamma_alt <- function(n, theta) {
  if (theta <= 0) stop("theta must be positive.")
  rgamma(n, shape = theta, rate = 1)
}

r_weibull_alt <- function(n, theta) {
  if (theta <= 0) stop("theta must be positive.")
  rweibull(n, shape = theta, scale = 1)
}

r_alt <- function(n, model = c("LFR", "Gamma", "Weibull"), theta) {
  model <- match.arg(model)
  switch(
    model,
    LFR = r_lfr(n, theta),
    Gamma = r_gamma_alt(n, theta),
    Weibull = r_weibull_alt(n, theta)
  )
}
```

S3.3. Null Critical Values, Empirical Size, and Power

```
## ------------------------------------------------------------
## Monte Carlo calibration and rejection probabilities
## ------------------------------------------------------------

null_statistics <- function(n, beta, B = 5000L) {
  replicate(B, T_beta_star(rexp(n, rate = 1), beta))
}

null_critical_value <- function(n, beta, alpha = 0.05, B = 5000L) {
  unname(quantile(null_statistics(n, beta, B), probs = 1 - alpha,
                  type = 8, na.rm = TRUE))
}
```

```
empirical_size <- function(n, beta, alpha = 0.05, B = 5000L,
                           critical_B = B) {
  cv <- null_critical_value(n, beta, alpha, critical_B)
  mean(replicate(B, T_beta_star(rexp(n, rate = 1), beta) > cv))
}

empirical_power <- function(n, beta, model, theta, alpha = 0.05,
                            B = 5000L, critical_B = B) {
  cv <- null_critical_value(n, beta, alpha, critical_B)
  mean(replicate(
    B,
    T_beta_star(r_alt(n, model = model, theta = theta), beta) > cv
  ))
}
```

S3.4. Sensitivity Study for Tables 4–6

```
## ------------------------------------------------------------
## Sensitivity study
## ------------------------------------------------------------

set.seed(20260623)

n_values <- c(20, 30, 40, 50)
beta_values <- seq(1.5, 5.0, by = 0.5)

theta_grid <- list(
  LFR = c(0.25, 0.75, 1.25, 1.75, 2.25),
  Gamma = c(1.2, 1.6, 2.0, 2.2, 2.6),
  Weibull = c(1.1, 1.3, 1.5, 1.7, 1.9)
)

run_sensitivity_table <- function(model, B = 5000L, alpha = 0.05) {
  grid <- expand.grid(
    n = n_values,
    theta = theta_grid[[model]],
    beta = beta_values,
    KEEP.OUT.ATTRS = FALSE
  )

  grid$power <- mapply(
    FUN = function(n, theta, beta) {
      empirical_power(
        n = n, beta = beta, model = model, theta = theta,
        alpha = alpha, B = B, critical_B = B
      )
    },
    n = grid$n, theta = grid$theta, beta = grid$beta
```

```
  )
  grid
}

table4_LFR <- run_sensitivity_table("LFR")
table5_Gamma <- run_sensitivity_table("Gamma")
table6_Weibull <- run_sensitivity_table("Weibull")

write.csv(table4_LFR, "Table4_LFR_sensitivity.csv", row.names = FALSE)
write.csv(table5_Gamma, "Table5_Gamma_sensitivity.csv", row.names = FALSE)
write.csv(table6_Weibull, "Table6_Weibull_sensitivity.csv", row.names = FALSE)
```

S3.5. Empirical p-Values for Observed Data

```
## ------------------------------------------------------------
## Monte Carlo p-value under the exponential null
## ------------------------------------------------------------

mc_pvalue <- function(x, beta, B = 100000L, seed = NULL) {
  if (!is.null(seed)) set.seed(seed)
  observed <- T_beta_star(x, beta)
  null_values <- replicate(
    B,
    T_beta_star(rexp(length(x), rate = 1), beta)
  )
  p_value <- (1 + sum(null_values >= observed)) / (B + 1)
  list(
    statistic = observed,
    p_value = p_value,
    beta = beta,
    n = length(x),
    B = B
  )
}
```

S3.6. Empirical MRL and Exponential Q-Q Diagnostics

```
## ------------------------------------------------------------
## Graphical diagnostics
## ------------------------------------------------------------

empirical_mrl <- function(x, t_values) {
  x <- as.numeric(x)
  vapply(t_values, function(t) {
    survivors <- x[x > t]
    if (length(survivors) == 0L) return(NA_real_)
    mean(survivors - t)
  }, numeric(1))
}
```

```
plot_exp_qq_and_mrl <- function(x, main_label = "Dataset") {
  x <- sort(as.numeric(x))
  n <- length(x)

  old_par <- par(no.readonly = TRUE)
  on.exit(par(old_par), add = TRUE)
  par(mfrow = c(1, 2), mar = c(4, 4, 3, 1))

  theoretical <- qexp(ppoints(n), rate = 1 / mean(x))
  qqplot(
    theoretical, x,
    main = paste(main_label, "- Exponential Q-Q Plot"),
    xlab = "Theoretical exponential quantiles",
    ylab = "Observed quantiles",
    pch = 19
  )
  abline(0, 1, lwd = 2)

  t_values <- seq(0, max(x) * 0.98, length.out = 60)
  mrl_values <- empirical_mrl(x, t_values)
  plot(
    t_values, mrl_values, type = "l", lwd = 2,
    xlab = "t", ylab = expression(hat(m)[n](t)),
    main = paste(main_label, "- Empirical MRL")
  )
  points(t_values, mrl_values, pch = 16, cex = 0.45)
  abline(h = mean(x), lty = 2)
}
```

S3.7. Data Used in the Three Applications

```
## ------------------------------------------------------------
## Real datasets
## ------------------------------------------------------------

data1 <- c(
  1.56, 8.51, 2.17, 0.37, 1.09, 9.84, 4.95, 3.18, 11.37, 2.81,
  6.22, 1.87, 9.05, 2.44, 1.38, 4.17, 3.74, 1.37, 2.33, 7.80,
  2.10, 0.47, 2.54, 4.92, 0.09, 0.18, 1.72, 1.02, 0.62, 2.34,
  0.50, 2.37, 3.65, 0.59, 5.76, 2.14, 0.88, 0.95, 4.17, 2.25
)

data2 <- c(
  10.49, 8.80, 12.42, 4.58, 6.85, 4.58, 5.00, 4.75, 4.75, 12.25,
  9.50, 13.54, 10.42, 4.65, 9.88, 6.21, 8.60, 7.06, 7.96, 7.89,
  9.70, 13.90, 12.65, 10.00, 12.65, 12.07, 9.80, 13.54, 9.82,
  13.54, 12.42, 12.73, 12.22, 12.25, 12.32, 8.75, 12.00, 17.50,
  11.88, 13.13, 13.56, 15.44, 13.22, 7.28, 11.70, 11.70, 11.60,
```

```
  10.90, 11.84, 8.00, 10.20, 5.77, 13.90, 4.58, 12.07, 15.44,
  10.20, 11.00, 8.50, 10.99, 10.39, 9.90, 13.94, 15.21, 13.56,
  9.00, 20.47, 15.22, 11.50, 13.90, 13.22, 10.48, 15.48, 9.80,
  12.21, 13.56, 7.04
)

data3 <- c(
  47.7, 50.2, 52.4, 52.5, 52.9, 53.8, 53.9, 54.6, 54.7, 54.9,
  55.3, 55.5, 56.4, 57.5, 59.0, 60.0, 61.1, 61.4, 62.4, 62.7,
  63.2, 63.5, 64.2, 65.4, 65.4, 65.6, 66.3, 66.6, 66.6, 66.8,
  67.2, 67.5, 67.6, 68.0, 68.4, 69.6, 70.4, 70.7, 72.6, 74.4
)

## Test results
set.seed(20260623)
lapply(list(data1 = data1, data2 = data2, data3 = data3), function(z) {
  list(
    beta_1.5 = mc_pvalue(z, beta = 1.5, B = 100000L),
    beta_2.0 = mc_pvalue(z, beta = 2.0, B = 100000L),
    beta_4.0 = mc_pvalue(z, beta = 4.0, B = 100000L)
  )
})

## Figures
plot_exp_qq_and_mrl(data1, "Dataset 1")
plot_exp_qq_and_mrl(data2, "Dataset 2")
plot_exp_qq_and_mrl(data3, "Dataset 3")
```

S3.8. Optional Parametric Model-Fitting Summaries

```
## ------------------------------------------------------------
## Exponential, Weibull, log-normal, and gamma fits
## Requires the recommended package MASS
## ------------------------------------------------------------
if (!requireNamespace("MASS", quietly = TRUE)) {
  stop("Install the MASS package before running this section.")
}
fit_candidates <- function(x) {
  x <- as.numeric(x)
  n <- length(x)
  fits <- list(
    exponential = MASS::fitdistr(x, densfun = "exponential"),
    weibull = MASS::fitdistr(x, densfun = "weibull"),
    lognormal = MASS::fitdistr(x, densfun = "lognormal"),
    gamma = MASS::fitdistr(x, densfun = "gamma")
  )
  cdf_fun <- list(
    exponential = function(q, est) pexp(q, rate = est[["rate"]]),
    weibull = function(q, est) pweibull(
```

```
      q, shape = est[["shape"]], scale = est[["scale"]]
    ),
    lognormal = function(q, est) plnorm(
      q, meanlog = est[["meanlog"]], sdlog = est[["sdlog"]]
    ),
    gamma = function(q, est) pgamma(
      q, shape = est[["shape"]], rate = est[["rate"]]
    )
  )
  rows <- lapply(names(fits), function(name) {
    fit <- fits[[name]]
    est <- fit$estimate
    logLik_value <- fit$loglik
    k <- length(est)
    ks <- suppressWarnings(ks.test(x, cdf_fun[[name]], est = est))
    data.frame(
      model = name,
      logLik = logLik_value,
      AIC = -2 * logLik_value + 2 * k,
      BIC = -2 * logLik_value + log(n) * k,
      KS = unname(ks$statistic),
      KS_pvalue = ks$p.value,
      row.names = NULL
    )
  })
  do.call(rbind, rows)
}
fit_candidates(data1)
fit_candidates(data2)
```
